# Crosstalk between SET7/9-dependent methylation and ARTD1-mediated ADP-ribosylation of histone H1.4

**DOI:** 10.1186/1756-8935-6-1

**Published:** 2013-01-05

**Authors:** Ingrid Kassner, Marc Barandun, Monika Fey, Florian Rosenthal, Michael O Hottiger

**Affiliations:** 1 Institute of Veterinary Biochemistry and Molecular Biology, University of Zurich, Winterthurerstrasse 190, Zurich, 8057, Switzerland; 2 Life Science Zurich Graduate School, Molecular Life Science Program, University of Zurich, Zurich, Switzerland

**Keywords:** PARP-1, SET7/9, Lysine methylation, Poly-ADP-ribosylation, Post-translational modification

## Abstract

**Background:**

Different histone post-translational modifications (PTMs) fine-tune and integrate different cellular signaling pathways at the chromatin level. ADP-ribose modification of histones by cellular ADP-ribosyltransferases such as ARTD1 (PARP1) is one of the many elements of the histone code. All 5 histone proteins were described to be ADP-ribosylated *in vitro* and *in vivo*. However, the crosstalk between ADP-ribosylation and other modifications is little understood.

**Results:**

In experiments with isolated histones, it was found that ADP-ribosylation of H3 by ARTD1 prevents H3 methylation by SET7/9. However, poly(ADP-ribosyl)ation (PARylation) of histone H3 surprisingly allowed subsequent methylation of H1 by SET7/9. Histone H1 was thus identified as a new target for SET7/9. The SET7/9 methylation sites in H1.4 were pinpointed to the last lysine residues of the six KAK motifs in the C-terminal domain (K121, K129, K159, K171, K177 and K192). Interestingly, H1 and the known SET7/9 target protein H3 competed with each other for SET7/9-dependent methylation.

**Conclusions:**

The results presented here identify H1.4 as a novel SET7/9 target protein, and document an intricate crosstalk between H3 and H1 methylation and PARylation, thus implying substrate competition as a regulatory mechanism. Thereby, these results underline the role of ADP-ribosylation as an element of the histone code.

## Background

Histones are nuclear proteins that package and order the DNA into nucleosomes [1]. Five major families of histones exist: H1 (H5), H2A, H2B, H3, and H4. Two copies of the core histones H2A, H2B, H3 and H4 form the octameric nucleosome core particles [2]. Unlike the other histones, only one copy of the linker histone H1 is present and stabilizes the DNA, which is wrapped around the core nucleosome [3]. Linker histones bind to both the nucleosome and the linker DNA region (approximately 20 to 80 nucleotides in length) between nucleosomes. The interaction of H1 with the nucleosome and additional DNA stretches at the entry/exit of the nucleosome forms the chromatosome and leads to higher order chromatin structure [4]. Many experiments addressing H1 function have been performed with purified, processed chromatin under low-salt conditions, but the *in vivo* role of H1 is less clear. Cellular studies have shown that overexpression of H1 can cause aberrant nuclear morphology and chromatin structure and, depending on the gene, H1 can serve as either a positive or a negative regulator of transcription [5]. Similar to the core histones, H1 is composed of three domains [6]. The N-terminus is a short, flexible segment rich in basic amino acids, the central domain exhibits a globular structure composed of a winged helix motif [6] and the C-terminus is predominantly composed of lysine, alanine and proline residues and is the main determinant for H1 binding to chromatin [7]. Among the five histone families of the chromatosome, the linker histone H1 is the least conserved. In the human genome, 11 genes encoding H1 variants have been identified and are transcribed either ubiquitously or in a cell type-specific manner [4,8]. The study described here focuses on histone H1.4, a histone variant that is expressed in somatic cells during S phase. Together with H1.2 it is the predominant histone variant in most cell types. Similar to the core histones, linker histones are subject to extensive post-translational modifications (PTMs), including phosphorylation, methylation and acetylation [9].

SET7/9 (also SET7, SET9, SETD7 or KMT7) is a mono-methyltransferase for the lysine residue at position 4 of histone H3 (H3K4) [10,11] that was linked to transcriptional activation. It methylates the consensus motif [K>R][S>KYARTPN][Kme] and prefers lysine residues within positively charged regions [12]. However, SET7/9 exhibits only weak lysine methyltransferase activity towards H3 in nucleosomes *in vitro*, suggesting that additional factors may affect SET7/9-dependent H3K4 methylation *in vivo*, or that histone proteins are not the main substrates of SET7/9. Lysine methylation can be reversed by demethylases of the lysine-specific demethylase (LSD) family or the Jumonji-C domain family of proteins [13,14].

In contrast to the canonical PTMs of the histone code, adenosine diphosphate (ADP)-ribosylation is much less studied. ADP-ribosylation comprises the transfer of the ADP-ribose moiety from the co-substrate nicotinamide adenine dinucleotide (NAD^+^) onto specific amino acid side chains of acceptor proteins or to pre-existing protein-linked ADP-ribose units by ADP-ribosyltransferases (ARTs). Mammalian ARTs can be divided into two groups according to their similarity to the bacterial diphtheria and cholera toxins - the ARTDs (also known as poly(ADP-ribose) polymerases (PARPs)) and ARTCs, respectively [15]. ARTD1 (PARP1) is the best-studied member of the ARTD family and represents a highly abundant (on average 1 × 10^6^ molecules per cell), chromatin-associated enzyme that is responsible for most (about 90%) of the cellular PAR generation [16,17]. It is implicated in many cellular processes such as the genotoxic stress response, cell cycle regulation, gene expression, differentiation and aging [18,19]. The major modification target of ARTD1 is ARTD1 itself, but it also modifies other nuclear proteins including all five histone proteins *in vitro* and *in vivo*[20]. In native chromatin, histone H1 is the main ADP-ribose acceptor, but depending on the chromatin composition and the accessibility of different histones, the ADP-ribosylation pattern of histones varies [21,22]. Mass spectrometry and electron-transfer dissociation (ETD) identified for the first time K13 of histone H2A, K30 of H2B, K27 and K37 of H3 as well as K16 of H4 as ADP-ribose acceptor sites (catalyzed by ARTD1) [23].

Crosstalk between different PTMs occurs directly by competition for acceptor sites or indirectly by changes in the accessibility of chromatin for modifying enzymes. The observation that specific lysine residues serve as ADP-ribose acceptors is important because the same amino acid residues are potential acetylation and methylation sites [24]. It is therefore likely that competition for acceptor sites between different histone PTMs such as ADP-ribosylation, acetylation, methylation and phosphorylation causes crosstalk [20]. This has been demonstrated by the finding that acetylation of lysine residue K16 of histone H4 inhibits ADP-ribosylation *in vitro*[23], which suggests that different crosstalk likely exists *in vivo* as well. Similarly, H1.4 K26 dimethylation and AuroraB-mediated phosphorylation of S27 have been reported to interfere with each other [25]. Whether or not other modifications of the histone code such as methylation or phosphorylation also crosstalk with ADP-ribosylation has not been studied before.

Here, we define the linker histone H1.4 as a novel target of SET7/9-dependent methylation, identify lysines K121, K129, K159, K171, K177 and K192 as methyl acceptor sites and describe crosstalk between H1.4 methylation and ADP-ribosylation as well as competition with histone H3 methylation.

## Results and discussion

### PARylation inhibits SET7/9-dependent methylation of histone H3

**Figure 1:**
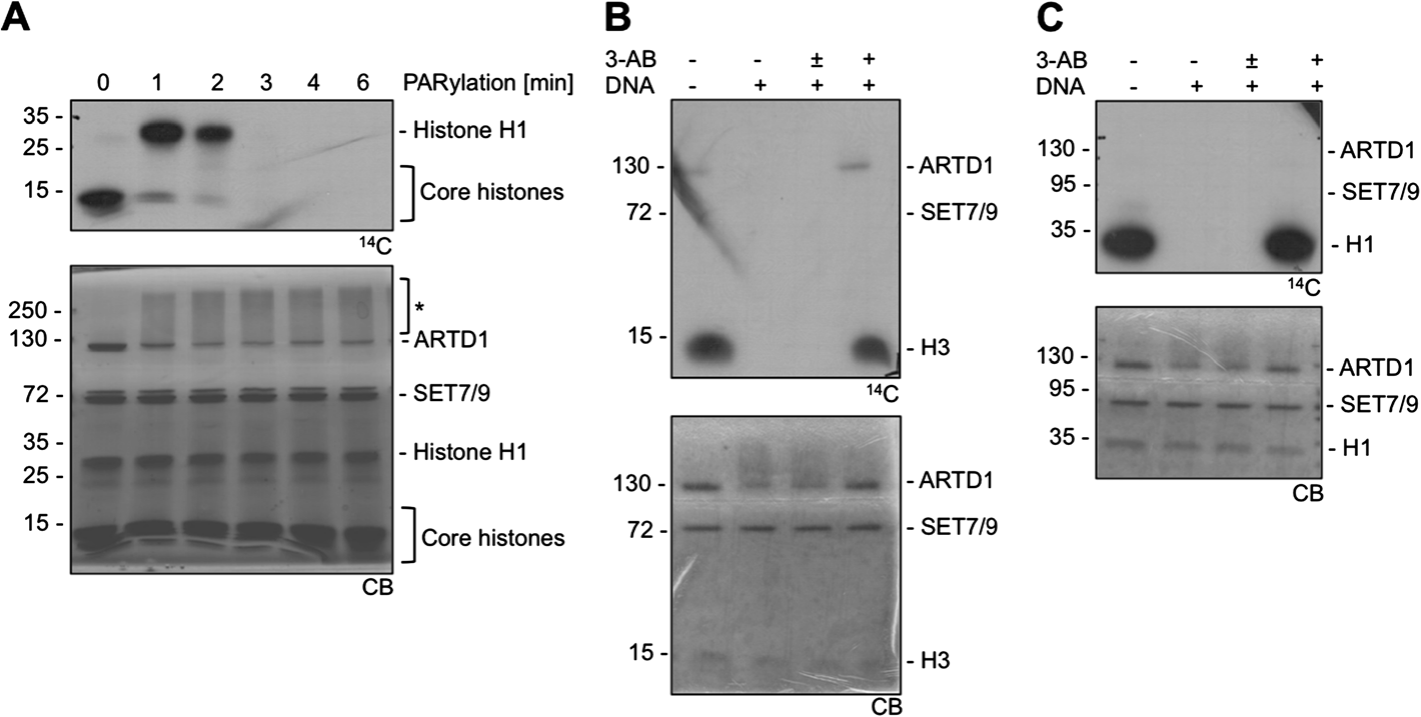
**PARylation inhibits SET7/9-dependent methylation of histone H3 and H1.** (**A**) ARTD1 was auto-modified for the indicated times and then inhibited by addition of PJ-34. A histone mix was added to the reaction either before or after the inhibition of ARTD1. Methylation was started after PJ-34 addition by adding SET7/9 and ^14^C-SAM. Proteins were separated by SDS-PAGE, stained with Coomassie blue (CB, lower blot) and methylation was analyzed by autoradiography (^14^C, upper blot). The asterisk marks the shift of automodified ARTD1. (**B**) H3 was incubated with ARTD1 and 160 μM NAD^+^. Activating DNA and PARP inhibitor 3-AB were present during the reaction as indicated. Methylation was then started by addition of SET7/9 and ^14^C-SAM ± 3-AB was added directly before addition of SET7/9 after the PAR reaction. (**C**) Influence of ARTD1 on H1 methylation as in (**B**). The approximate molecular weight is indicated on the left of each gel/blot.

ADP-ribosylation is a PTM of a wide variety of target proteins, including histones [20,23,26]. However, since histone tails are subject to many types of PTMs, crosstalk between different modifications likely exists. Therefore, it was investigated whether histone PARylation by ARTD1 affects consecutive SET7/9-dependent H3 methylation. A histone mix was PARylated for different time periods and then subjected to methylation assays with SET7/9 and radio-labelled S-adenosyl-L-(methyl-^14^C) methionine (^14^C-SAM) as methyl donor. ARTD1 and histones were both strongly PARylated and the level of modification correlated with the reaction times (see Additional file 1). In the absence of PARylation, mainly core histones, which comprise H3, were methylated (time-point 0) (Figure 1A). However, after 1 minute of PARylation of the histones by ARTD1, the methylation of core histones was already strongly reduced.

As a control for these results, recombinant H3 was incubated with ARTD1, NAD^+^ and with or without DNA (to stimulate ARTD1) and 3-AB (to inhibit ARTD1). H3 was methylated by SET7/9 if ARTD1 was inactive or inhibited (Figure 1B, lanes 1 and 4), but prevented in the samples containing activated ARTD1 (Figure 1B, lanes 2 and 3). PARylation of SET7/9 was not the cause for this effect, because the methyltransferase was only added after the PARylation reaction and addition or omission of 3-AB did not affect the result (Figure 1B, lanes 2 and 3).

### PARylation of histone H3 allows methylation of H1 by SET7/9

Interestingly, ADP-ribosylation of H3 for 1 or 2 minutes and subsequent inhibition of its methylation resulted in an unexpected and strong H1 methylation (Figure 1A). In order to confirm H1 methylation by SET7/9, experiments with purified H1 were performed. As before, H1 was incubated with ARTD1, NAD^+^ and with or without DNA and 3-AB. Similar to H3, histone H1 was methylated by SET7/9 in the absence of active ARTD1, but H1 methylation was completely inhibited by PARylation (Figure 1C). These results suggested that in addition to H3, histone H1 is a new methylation target of SET7/9 and that prior PARylation of H1 and H3 prevents consecutive methylation by SET7/9.

### Histones H1 and H3 compete for SET7/9-dependent methylation

**Figure 2:**
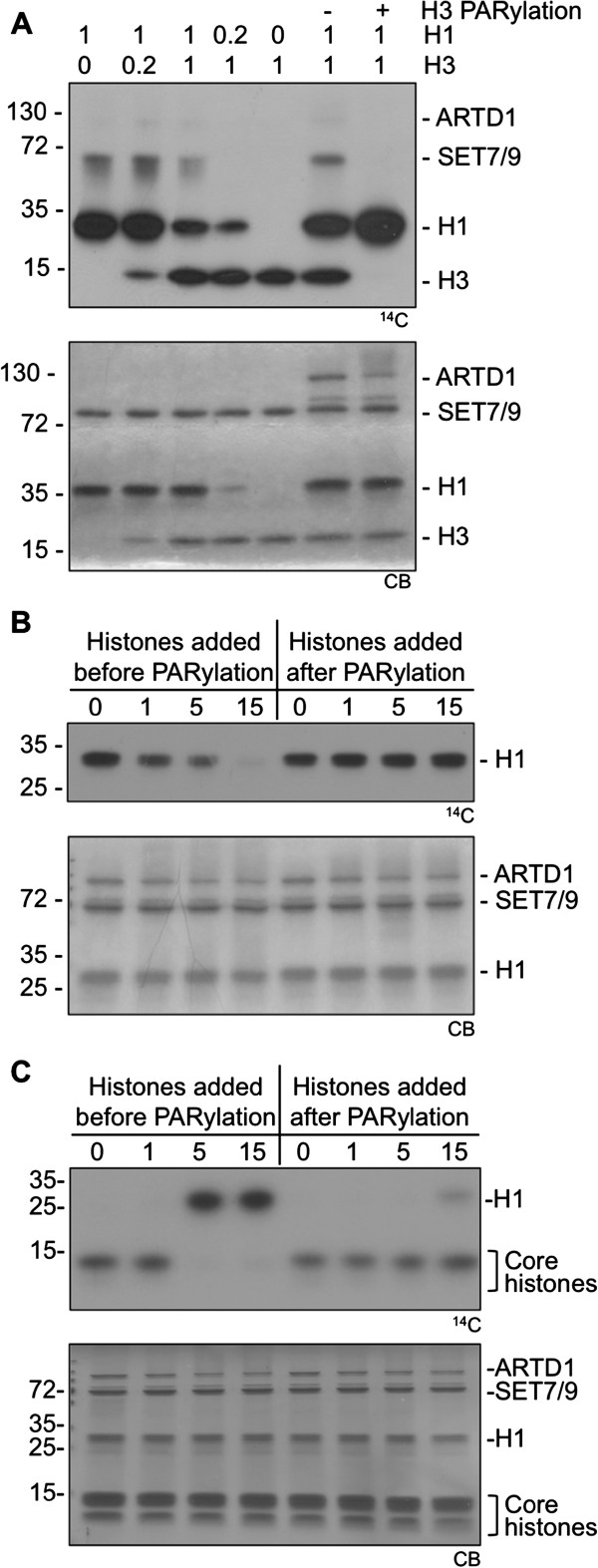
**Histones H1 and H3 compete for SET7/9-dependent methylation.** (**A**) H3 and H1 compete for methylation by SET7/9. H1 was methylated in presence of the indicated molar ratios of H3. In lanes 6 and 7, H3 was incubated with ARTD1 and NAD^+^ in the presence or absence of 3-AB before addition of H1 and the methylation reaction. (**B**) ARTD1 was auto-modified for the indicated times and then inhibited by addition of PJ-34. H1 was added to the reaction either before or after the PARylation. Methylation was started after addition of PJ-34 by adding SET7/9 and ^14^C-SAM. (**C**) As for (**B**), but a histone mix was used.

The results presented so far suggested that H1 and H3 are both methylated by SET7/9, but only in the absence of PARylation. ARTD1-dependent PARylation might thus modulate which histone protein is methylated. To test this hypothesis, *in vitro* competition experiments with or without PARylation of H1 and H3 were performed. H1 and H3 were strongly methylated by SET7/9 if present alone (Figure 2A, lanes 1 and 5). However, H1 and H3 competed with each other when present in the same reaction and thus lead to a reduced methylation signal (lanes 2 to 4). Prior PARylation of histone H3 completely abolished its competing activity for SET7/9-dependent H1 methylation (lanes 6 to 7). To further study and confirm this finding, a PARylation time course experiment with histone H1 or histone mix prior to SET7/9 methylation was performed. Set7/9-dependent methylation of H1 was abolished after 15 minutes of PARylation by ARTD1 (Figure 2B). In contrast, H3 methylation in a histone mix was already inhibited after 5 minutes of ARTD1 treatment, which lead to consecutive H1 methylation by Set7/9 (Figure 2C). In both cases, addition of the histones after the PARylation reaction did not influence consecutive Set7/9-dependent methylation (that is, H1 was methylated only if H3 was not present).

These results suggested that, in comparison to H1, H3 is preferentially modified by both, SET7/9 and ARTD1. In the short window when H1 methylation is inhibited due to PARylation but H3 is not yet fully ADP-ribosylated, SET7/9 dependent methylation of H3 is detectable. This example thus illustrates how different affinities for modifying enzymes can induce a switch of target proteins. These results suggest that differential PARylation of histone proteins by ARTD1 can indirectly influence histone methylation and thus the histone code by determining which target proteins are modified. In addition to crosstalk between PARylation and methylation, SET7/9 function is thus also subject to crosstalk between different substrates (for example, H3 and H1).

### The C-terminus of linker histone H1.4 is methylated by SET7/9

**Figure 3:**
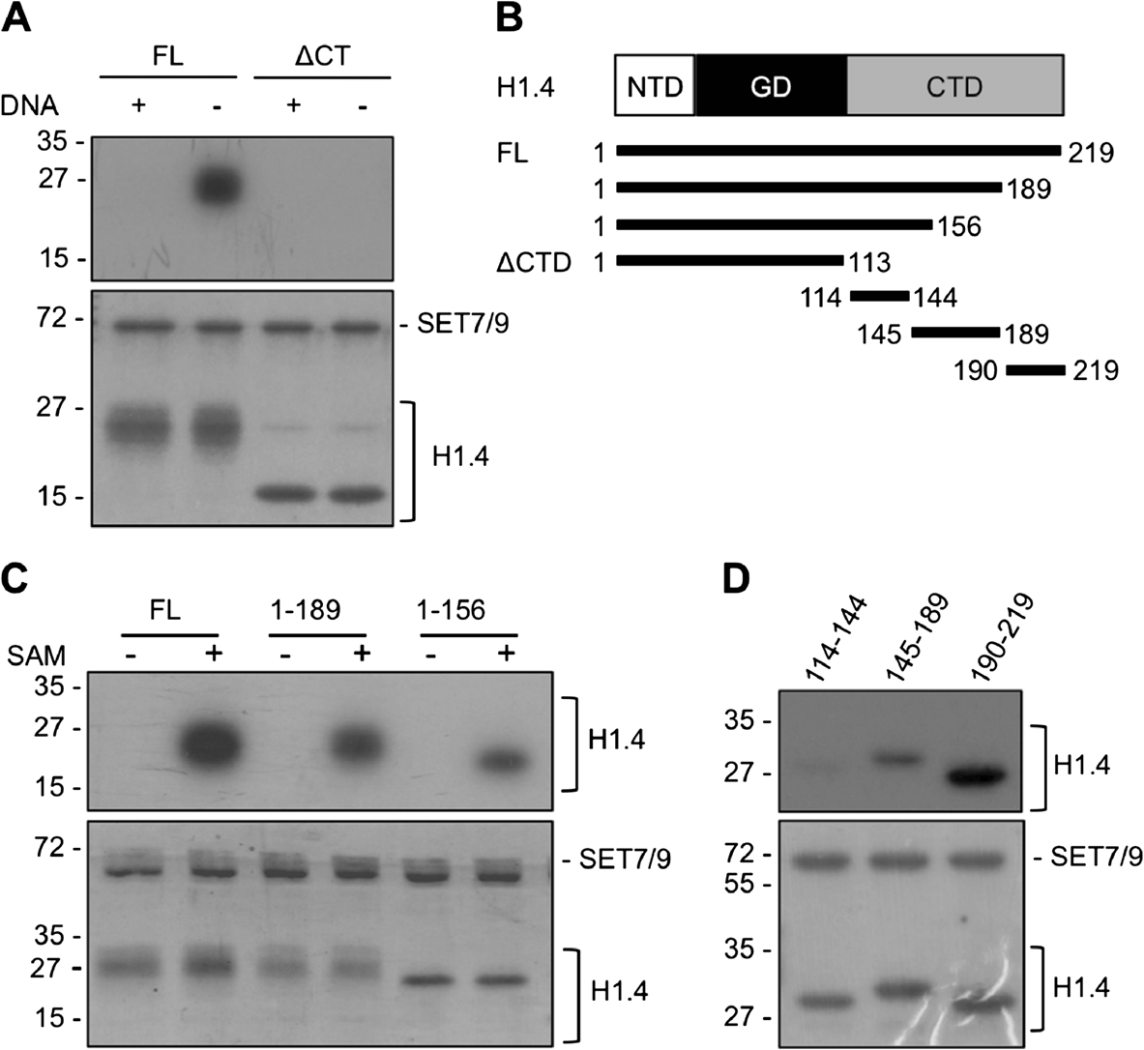
**Linker histones are methylated by SET7/9.** (**A**) HIS-tagged H1.4 FL and a deletion mutant without the CTD (ΔCTD) were *in vitro* methylated by SET7/9 in the presence or absence of plasmid DNA. (**B**) Schematic depiction of H1.4 wild-type (WT) protein and constructs. NTD, N-terminal domain; GD, globular domain; CTD, C-terminal domain; ΔCT, H1.4 construct completely lacking the C-terminus. (**C**) Recombinant HIS-tagged H1.4 full-length (FL) and deletion mutants were methylated *in vitro* by SET7/9. (**D**) H1.4 C-terminal fragments were expressed as GST fusion proteins and methylated *in vitro* by SET7/9. Methylation was analyzed by autoradiography.

In order to characterize the new SET7/9 methylation target H1, different fragments of H1.4 were created and their methylation was analyzed. Interestingly, full-length histone H1.4 was not methylated in the presence of plasmid DNA and neither was an H1.4 mutant lacking the C-terminus (ΔCT) (Figure 3A). These results suggested that H1.4 is methylated at the C-terminal domain (CTD), which has been reported to bind to DNA, and that SET7/9 likely methylates soluble H1.4 that is not part of the chromatin structure. In order to locate the methylation site in histone H1.4, recombinant, C-terminally truncated, HIS-tagged H1.4 fragments as well as short fragments of the CTD were analyzed (Figure 3B). Full length H1.4 (aa 1 to 219) was strongly methylated, while partial truncation of the CTD (1 to 189 or 1 to 156) caused reduced SET7/9-dependent methylation (Figure 3C). To further pinpoint the methylation sites in the H1.4 CTD, three H1.4 fragments covering the whole CTD were expressed as glutathione-S-transferase (GST)-fusion proteins (aa 114 to 144, 145 to 189, 190 to 219, Figure 3B) and used in methylation assays. All three fragments were methylated by SET7/9, albeit to different degrees (Figure 3D). Fragment 190 to 219 showed the strongest methylation, while the fragment from amino acids 114 to 144 was only weakly methylated, suggesting that multiple SET7/9-dependent methylation sites are present in the CTD of H1.4. These results demonstrated that the linker histone H1.4 is methylated by SET7/9 *in vitro*.

## Linker histone H1.4 is methylated at six lysine residues of the C-terminal domain

In order to define all SET7/9-dependent methylation sites in the CTD of the H1.4, the C-terminal fragments (114 to 144, 145 to 189, 190 to 219) were further mutagenized. Since the strongest methylation was seen for fragments 145 to 189 and 190 to 219, these peptides were subjected to a cluster mutation approach. Seven lysine clusters that comprised all potential methylation sites were defined and in each of these clusters all lysine residues were mutated to arginines (Additional file 2, Figure S2A). Mutation of cluster 4 and cluster 6 did not interfere with the methylation of the H1.4 fragments, indicating that the lysine residues located in these two clusters are not methylated by SET7/9 (Additional file 2: Figure S2B). The remaining cluster mutants 1, 2, 3, 5 and 7 were less methylated than the wild-type (WT) proteins (Additional file 2: Figure S2B). Computational analysis of these clusters as well as the fragment 114 to 144 identified six putative SET7/9 target sites matching the (KR)(STA)K consensus motif for SET7/9-dependent methylation [27]. These lysines at position 121, 129, 159, 171, 177 and 192 represent the last residues of the six KAK motifs in the CTD of H1.4 (Additional file 2, Figure S2A).

**Figure 4:**
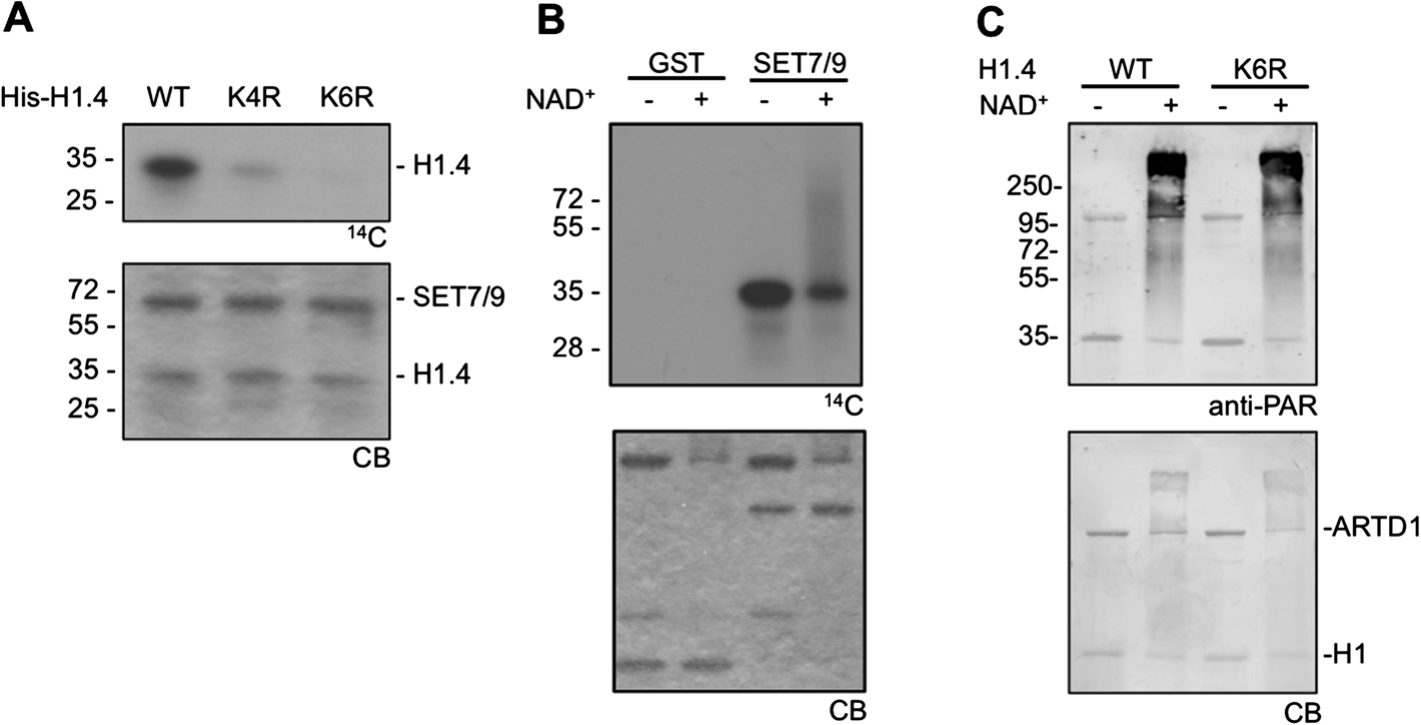
**Full-length H1.4 is methylated at six lysine residues by SET7/9.** (**A**) Mutation of the four lysine residues K159, K171, K177 and K192 (K4R) in wild-type (WT) H1.4 reduces SET7/9-dependent methylation. The additional mutation of K121 and K129 (K6R) abolishes methylation of full-length H1.4 by SET7/9. (**B**) H1.4 is efficiently PARylated after methylation by SET7/9. H1.4 was first incubated with S-adenosyl-L-(methyl-^14^C) methionine (^14^C-SAM) and glutathione-S-transferase (GST) or SET7/9 and then incubated with ADP-ribosyltransferase-D1 (ARTD1) in the presence or absence of cold nicotinamide adenine dinucleotide (NAD)^+^. (**C**) H1.4 K6R is still modified by ARTD1. H1.4 WT and K6R were incubated with ARTD1 in the presence of 250 μM NAD^+^. PARylation was analyzed by Western blotting.

The corresponding lysine residues were then mutated individually and in combination. Only the mutation of all three lysines (K159, K171, K177) of the fragment 145 to 189 completely abolished the methylation of this peptide (Additional file 2, Figure S2C). Similarly, the K192R mutation abrogated the SET7/9-dependent methylation of fragment 190 to 219 (Additional file 2, Figure S2D). Interestingly, the combination of all four mutations (K159R, K171R, K177R and K192R) did not completely prevent the methylation of full-length H1.4 (Figure 4A). The additional mutation of K121 and K129 of the fragment 114 to 144 to arginines reduced the SET7/9-dependent methylation of full-length H1.4 most significantly, although a faint signal was sometimes observed even for this peptide (Figure 4A).

In summary, the mapping of the methylation sites in the H1.4 CTD thus identified the KAK motif and the six lysines K121, K129, K159, K171, K177 and K192 as the targets for SET7/9-dependent methylation of this linker histone variant.

## Histones H1.4 and H3 methylation does not affect PARylation

The initial observation revealing H1 methylation by SET7/9 indicated crosstalk between PARylation and methylation as well as competition between H3 and H1.4. In order to elucidate the nature of this crosstalk, the influence of methylation on PARylation was studied and additional competition experiments with H1.4 and H3 were performed.

Prior methylation by SET7/9 did not prevent consecutive H1.4 PARylation, as observed by the shift of methylated H1 (Figure 4B). Similarly, the non-methylated H1.4 K6R mutant and WT H1.4 were modified by ARTD1 comparably (Figure 4C). These results clearly indicated that PARylation and SET7/9-dependent methylation of H3 and H1.4 do not crosstalk due to the same modification sites, even though prior PARylation does prevent methylation (Figures 2B, 2C). It is therefore likely that PARylation inhibits SET7/9 interaction with PARylated H3 or H1.4 and thereby prevents methylation.

## Conclusions

This study describes the linker histone H1.4 as a new target for the H3K4 mono-methyltransferase SET7/9. Full-length histone H1.4 was methylated by SET7/9 at the lysine residues K121, K129, K159, K171, K177 and K192 of the KAK motifs of the CTD, which suggests a strong preference of SET7/9 for this recognition sequence. However, some of the KAK motifs seemed preferentially methylated, which may indicate a sequential modification or hint at differences in the accessibility of the different methylation sites.

The addition of plasmid DNA abolished SET7/9-dependent methylation of target proteins, indicating that incorporation into the nucleosome structure and DNA binding prevents methylation. This could be explained by DNA-induced conformational changes of the H1 CTD and by charge neutralization through DNA, as SET7/9 is known to prefer lysine residues in a positively charged context. Likewise, methylation may directly influence the binding of H1 to DNA and its function in chromatin compaction, especially as the six lysine residues targeted by SET7/9 all reside in the CTD of H1.4, which is important for chromatin binding [28]. These findings may indicate that SET7/9-dependent H1 methylation appears when H1 is not chromatin bound and possibly influences its turnover and exchange, which extends the scope of the histone code to the extra-chromatin realm.

**Figure 5:**
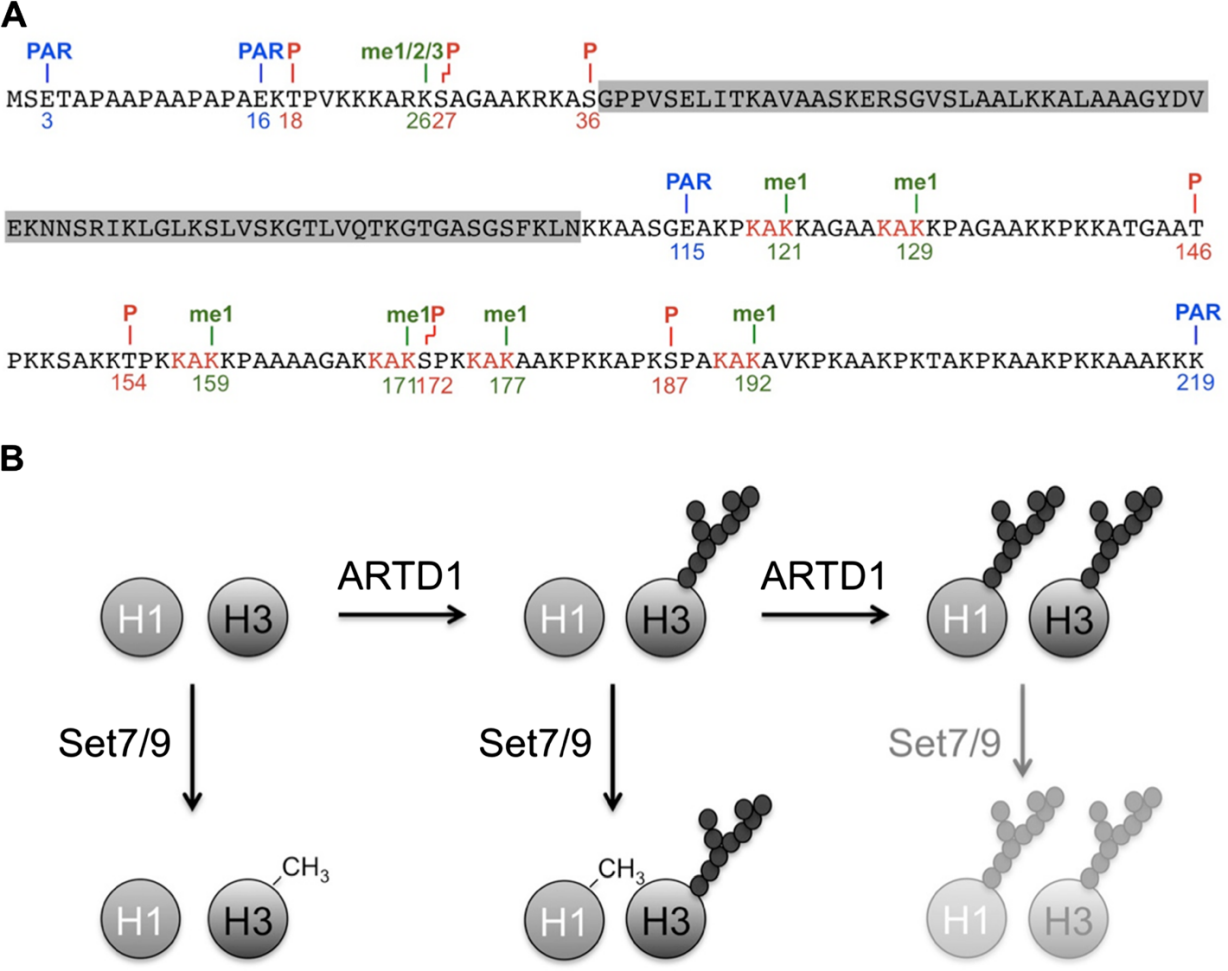
**Crosstalk between adenosine diphosphate-ribosyltransferase (ARTD)-1 and SET7/9-dependent modification of linker and core histones.** (**A**) Summary of known post-translational modification sites of H1.4. The grey box marks the central globular domain. The six newly identified KAK motifs that are methylated by SET7/9 are highlighted in red. Blue: PARylation (PAR), red: phosphorylation (P), green: methylation (me, number indicates methylation state). (**B**) Model of the sequential adenosine diphosphate (ADP)-ribosylation and methylation reactions on histone H3 and H1 by ARTD1 and SET7/9.

Covalent histone modifications can alter chromatin structure and thereby define transcriptionally active or inactive chromatin states [29]. Compared with core histones, little is known about the modifications and the corresponding modifiers of the linker histones. One possible function of H1 methylation by SET7/9 might be the stimulation or repression of other post-translational modifications. Crosstalk between K26 dimethylation and AuroraB-mediated phosphorylation of S27 has been reported previously [25]. In addition, the H1.4 CTD is the target of acetylation, ADP-ribosylation and phosphorylation [30,31]. Interestingly, the four last KAK motifs, which displayed the strongest methylation *in vitro*, are located in close proximity to well known CDK phosphorylation sites (Figure 5A), which suggests a potential for significant crosstalk between acetylation, methylation and phosphorylation. Two of the sites in this region, S172 and S187, are not exclusively phosphorylated during mitosis and their phosphorylation in interphase correlated with transcription and chromatin relaxation [32]. Crosstalk between different histone modifications of H1.4 thus can fulfill important cellular functions. However, in most cases the modifying enzymes are currently not known and thus the biological and cellular functions of these modifications have not been elucidated in detail yet.

ARTD1 and histone ADP-ribosylation were previously suggested as components of the histone code [20,26]. The results described here provide a further line of evidence for this hypothesis. We show that ARTD1-dependent PARylation of histones influences their subsequent methylation by SET7/9. Compared to phosphorylation, which strongly reduces methylation if in proximity of SET7/9 target lysine residues [12], PARylation is a much more bulky modification with more negative charges and therefore also inhibited histone methylation by SET7/9. Based on the experiments shown here, SET7/9 methylates both H1 and H3, but prefers H3 if both substrates are present. Strikingly, PARylation did not merely inhibit SET7/9-dependent methylation of histones, but shifted its target from H3 to H1 (Figure 5B). These observations could be explained by sequential PARylation events, where ARTD1 modifies H3 before H1. PARylation of H3 would then inhibit its subsequent methylation by SET7/9 and its competition as a SET7/9 target with H1.

This ARTD1-dependent regulation of the substrate specificity of a histone modifying enzyme may be an exciting mechanism to explain how ARTD1 influences chromatin-associated processes such as transcription. It is also important to note that progressive PARylation of histones also inhibited the methylation of H1, documenting that different outcomes for SET7/9-dependent histone methylation can occur depending on the extent of ARTD1 activity.

The complexity of ARTD1-dependent histone modifications is increased by the facts that (1) ARTD1 can modify all core histones and the linker histones [23], and (2) the size and quality (for example, branching or length) of the polymers may differ under varying conditions and depending on the substrate histone. Therefore, future studies should not merely focus on the influence of ARTD1 and PARylation on other histone modifications but also on how ARTD1-dependent histone modification itself is regulated. In this regard, it will also be interesting to study specific ADP-ribose modifications and their effect on SET7/9 methylation. Although we have identified ADP-ribose acceptor sites in histones (for example, K37 of H3) it is not yet possible to chemically synthesize peptides with specific ADP-ribose modifications required for these studies. Furthermore, antibodies specific for a particular ADP-ribose modification have not been developed. In the future, such tools will allow study of the effect of SET7/9-dependent methylation on ARTD1 activity and modification sites in much more detail.

## Methods

### Plasmids and protein expression

pGEX-SET7/9 (52 to 366) and pET28b-H1.4 (fl) bacterial expression vectors were kind gifts from D Reinberg and R Schneider, respectively. H1.4 full-length and deletions mutants were subcloned into pET-28a to add the N-terminal HIS-tag. C-terminal H1.4 fragments were cloned into pGEX6P1. All point mutations were inserted by site-directed mutagenesis. Cluster mutants were created by overlapping PCR with the corresponding primers.

The baculovirus expression vector BacPak8 (Clontech, Mountain View, CA, USA) was used for the expression of recombinant ARTD1 in Sf21 insect cells, as described previously [33]. GST- and HIS-tagged histone proteins were expressed in *E.coli*. All recombinant proteins were purified by a one-step affinity chromatography using ProBond resin (Invitrogen, Paisley, UK) for HIS-tagged and glutathione sepharose (GE Healthcare, Uppsala, Sweden) for GST-tagged proteins, according to the manufacturer‘s recommendations. HIS-H1.4(fl) proteins were purified as described elsewhere [34].

### Reagents

Lyophilized histones or H1 mix from calf thymus were purchased from Roche (Rotkreuz, Zug, Switzerland) and resolubilized in water. The 3-AB (Sigma-Aldrich, St. Louis, MO, USA) was freshly prepared in water.

### *In vitro* methylation assays

If not stated otherwise, approximately 1 μg histone proteins were incubated with 1 μg bacterially purified GST-SET7/9 in the presence of 0.03 μCi ^14^C-SAM (PerkinElmer, Boston, MA, USA) in methylation buffer (50 mM Tris–HCl pH8.0, 50 mM NaCl, 10% glycerol, 1 mM PMSF, 1 mM DTT) for 10 to 60 minutes at 30°C in a 25 μl reaction. For DNA inhibition assays, histones were preincubated with or without 0.5 μg plasmid DNA (pcDNA) and then methylated for 15 minutes. Reactions were stopped by addition of 10× SDS-loading buffer, boiled and separated by SDS-PAGE. Gels were stained with CB, incubated in 1 M sodium salicylate for 20 minutes, dried, and exposed on x-ray film at −80°C.

### Sequential ADP-ribosylation and methylation assays

Sequential modification assays were performed in PAR buffer (50 mM Tris–HCl, 50 mM NaCl, 4 mM MgCl_2_, 250 μM DTT, 1 mg/ml pepstatin, 1 mg/ml bestatin, 1 mg/ml leupeptin).

We incubated 10 pmol ARTD1, with or without 2.5 μg histone mix or 1 μg individual histones, in the presence or absence of 5 pmol activating DNA and 160 μM NAD^+^ (Sigma-Aldrich, St. Louis, MO, USA) for 15 minutes at 30°C; 8 mM 3-AB or 0.2 mM PJ-34 were added to inhibit ARTD1 activity either before the ADP-ribosylation or afterwards, where indicated. The methylation was then started by addition of 1 μg SET7/9 and 0.03 μCi ^14^C-SAM and allowed to proceed for 1 h at 30°C. Autoradiography was performed as described above. The activating DNA used in all assays was an annealed double-stranded oligomer (5’-GGAATTCC-3’).

## Abbreviations

ADP: Adenosine diphosphate; ART: Adenosine diphosphate ribosyltransferase; CB: Coomassie blue; C-SAM: S-adenosyl-L-(methyl-^14^C) methionine; CTD: C-terminal domain; ETD: Electron-transfer dissociation; GST: Glutathione-S-transferase; LSD: Lysine-specific demethylase; NAD: Nicotinamide adenine dinucleotide; PARP: Poly(ADP-ribose) polymerase; PCR: Polymerase chain reaction; PTM: Post-translational modification; WT: Wild-type.

## Competing interests

The authors declare that they have no conflicts of interest.

## Authors’ contributions

IK and MOH designed the experiments; IK, MB, FR and MF performed experiments; MOH designed and supervised the study. IK and MOH wrote the manuscript. All the authors read and agreed with the manuscript.

## Supplementary Material

Additional file 1 **Figure S1.** Time-dependent PARylation of adenosine diphosphate-ribosyltransferase (ARTD)-1 and histones. ARTD1 and a histone mix were modified for the indicated times. Proteins were separated by SDS-PAGE, stained with Coomassie blue (CB, upper blot) and adenosine diphosphate (ADP)-ribosylation was analyzed by autoradiography (^32^P, lower blots showing a long and short exposure). The mobility shift of poly-ADP-ribosylated ARTD1 is marked in the SDS-PAGE gel. Click here for file

Additional file 2 **Figure S2.** H1.4 C-terminal domain (CTD) is methylated at KAK* motifs. **(A)** Amino acid sequences of C-terminal H1.4 fragments. Boxes represent the lysine clusters which were mutated to arginines in (B). Grey boxes mark the clusters that markedly influence methylation. KAK motifs are highlighted in dark grey and the methylated lysines are bold. **(B)** Cluster mutant approach to identify methylation sites in H1.4 CTD, which contains 43 lysine residues as potential target sites. Clusters were mutated one by one in the corresponding C-terminal fragment and methylation efficiency by SET7/9 was tested *in vitro*. **(C)** Methylation of C-terminal fragments by SET7/9 was tested *in vitro* after mutation of single lysine residues in KAK* motifs. **(D)** Methylation of H1.4 (145–189) double and triple mutants by SET7/9. Click here for file
