# Supplementary figures and images for: Crosstalk between SET7/9-dependent methylation and ARTD1-mediated ADP-ribosylation of histone H1.4
Source: Epigenetics Chromatin. 2013 Jan 5;6:1. doi: 10.1186/1756-8935-6-1 (PMC3554541; doi:10.1186/1756-8935-6-1)

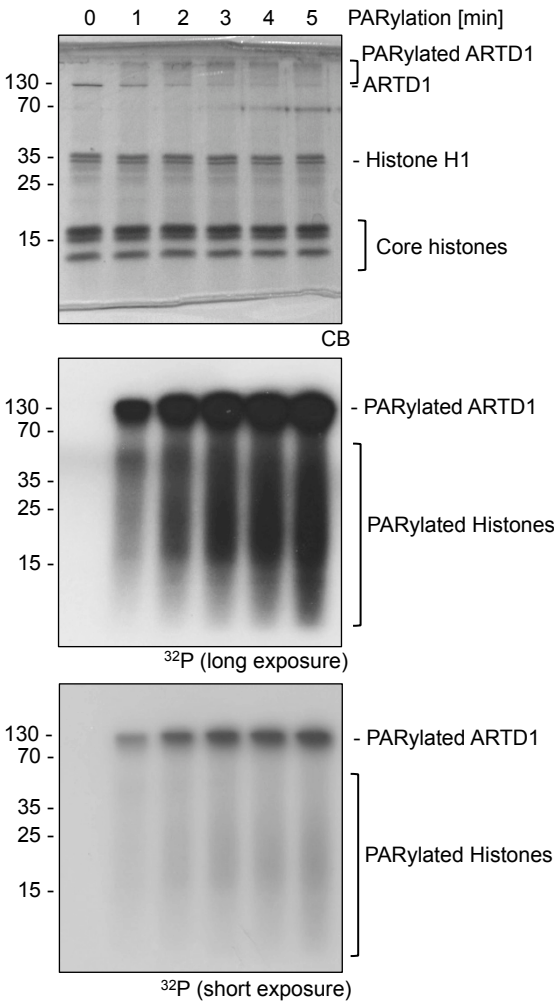

Supplement: Additional file 1 — Figure S1. Time-dependent PARylation of adenosine diphosphate-ribosyltransferase (ARTD)-1 and histones. ARTD1 and a histone mix were modified for the indicated times. Proteins were separated by SDS-PAGE, stained with Coomassie blue (CB, upper blot) and adenosine diphosphate (ADP)-ribosylation was analyzed by autoradiography (32P, lower blots showing a long and short exposure). The mobility shift of poly-ADP-ribosylated ARTD1 is marked in the SDS-PAGE gel. [file 1756-8935-6-1-S1.pdf]

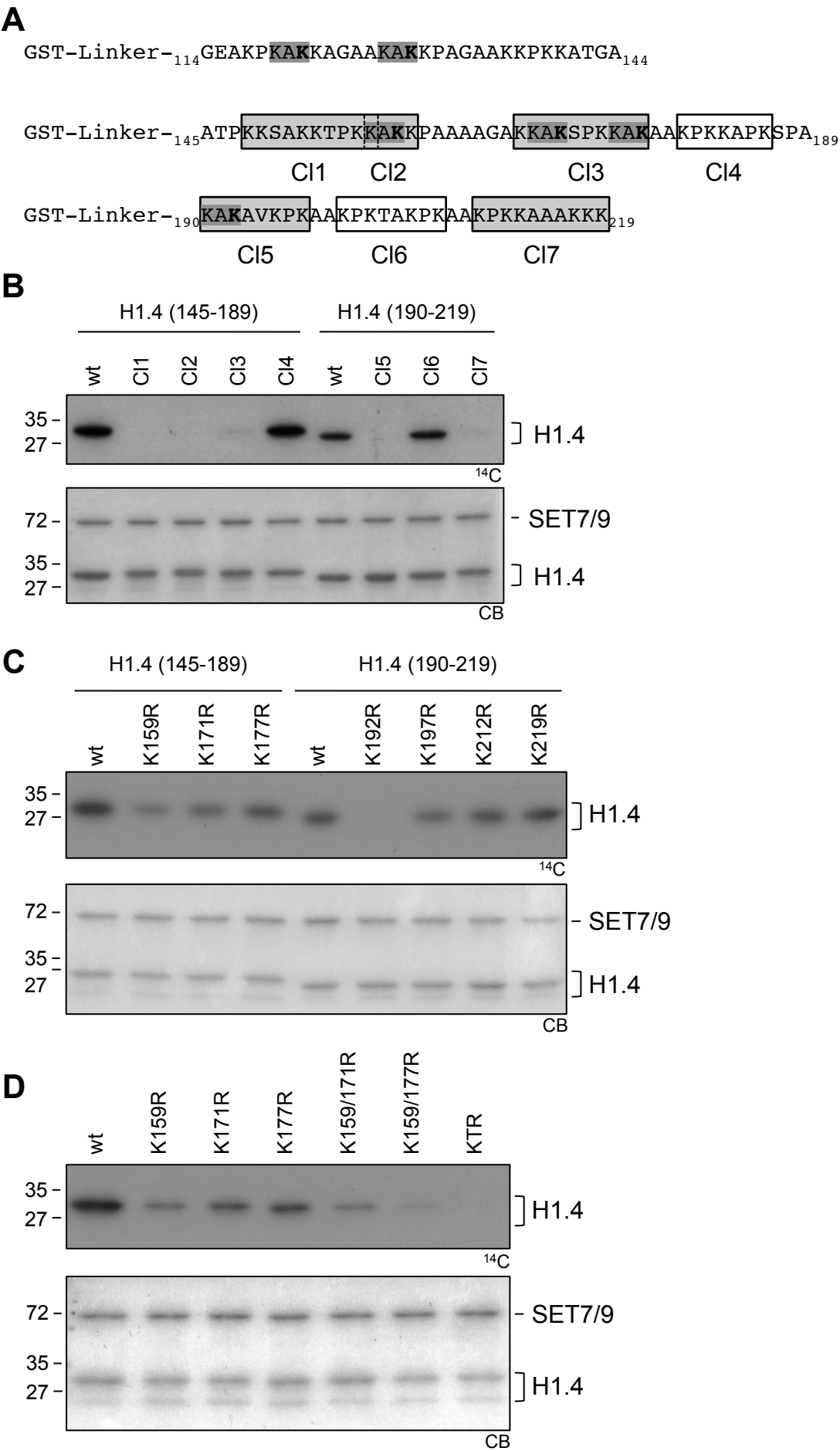

Supplement: Additional file 2 — Figure S2. H1.4 C-terminal domain (CTD) is methylated at KAK* motifs. (A) Amino acid sequences of C-terminal H1.4 fragments. Boxes represent the lysine clusters which were mutated to arginines in (B). Grey boxes mark the clusters that markedly influence methylation. KAK motifs are highlighted in dark grey and the methylated lysines are bold. (B) Cluster mutant approach to identify methylation sites in H1.4 CTD, which contains 43 lysine residues as potential target sites. Clusters were mutated one by one in the corresponding C-terminal fragment and methylation efficiency by SET7/9 was tested in vitro. (C) Methylation of C-terminal fragments by SET7/9 was tested in vitro after mutation of single lysine residues in KAK* motifs. (D) Methylation of H1.4 (145–189) double and triple mutants by SET7/9. [file 1756-8935-6-1-S2.pdf]
